# Rapid Liquid Chromatography–Tandem Mass Spectrometry Method for Determination of Total and Free Testosterone in Human Serum and Its Application to Monitoring Biomarker Response of Elite Athletes

**DOI:** 10.3390/molecules29215007

**Published:** 2024-10-23

**Authors:** Jianli Zhang, Hang Yu, Yulin Shen, Xingya Yang, Yan Wang

**Affiliations:** 1 China Institute of Sport Science, General Administration of Sport of China, Beijing 100061, China; shenyulin@ciss.cn (Y.S.); yangxingya@ciss.cn (X.Y.); 2 State Key Laboratory of Bioactive Substance and Function of Natural Medicines, Institute of Materia Medica, Chinese Academy of Medical Sciences & Peking Union Medical College, Beijing 100050, China; yuhang@imm.ac.cn (H.Y.); wangyan@imm.ac.cn (Y.W.)

**Keywords:** total testosterone, free testosterone, LC-MS/MS, biomarker, athlete

## Abstract

Total testosterone (TT) and free testosterone (FT) are important biochemical markers for anabolism of the human body, and can also serve as early screening indicators for overtraining syndrome (OTS). Presently, there is no fast and reliable serum TT and FT determination method in the field of sport science that can meet the requirements of sports research. Thus, a rapid and accurate determination method for serum TT and FT to fill the gap is needed urgently in sports training. Herein, a simple and reliable liquid chromatography–tandem mass spectrometry (LC-MS/MS) method for the simultaneous determination of TT and FT in serum was developed and fully validated, followed by the application of professional athletes in training monitoring. Efficient pretreatments based on only one-step liquid–liquid extraction (LLE) for TT and one-step LLE after a 20 min ultrafiltration for FT were adopted in this study, and the isotope internal standard of testosterone-13C3 was used to ensure the reliability of the whole procedure. A linear range of four orders of magnitude with 0.02–100 ng/mL can meet the concentration range requirement between a higher limit for male TT and a lower limit for female FT. The accuracy, precision, stability, and matrix effect were all within the limits of the guidelines. The serum TT and FT levels of 200 professional athletes (98 male athletes and 102 female athletes) were investigated by this method. Serum TT, FT, and FT/TT levels of professional athletes were significantly higher than the general population, and serum TT levels were significantly higher by LC-MS/MS than by a chemiluminescence immunoassay. In conclusion, the LC-MS/MS method for TT and FT measurement developed in this study is time-saving and easy to operate, which can be used as a reliable method for the determination of serum TT and FT in sports training, offering valuable information for monitoring anabolism of athletes and screening OTS in the early stage.

## 1. Introduction

In competitive sports, monitoring biomarker responses to training may inform changes in training load to optimize performance and enhance positive adaptation with the reduction in injury risk for elite athletes. As an anabolic hormone with multiple physiological functions in the human body, testosterone (T) plays an important role in the growth and maintenance of skeletal muscle, bone, and red blood cells [1,2]. Therefore, it can be used as a biomarker to show evidence of some changes in direction, which corresponds to a change in performance, thus indicating the potential for tracking performance [3,4,5,6]. In addition, it can also provide some emotional assistance, such as reducing fear-potentiated startle [7].

In the circulation, T is bound tightly to sex hormone-binding globulin (SHBG), approximately 44% in men and 66% in women; a small fraction is bound weakly to human serum albumin (HAS), corticosteroid-binding globulin (CBG), and orosomucoid (ORM); only 1% to 4% of circulating T is unbound or free [8,9,10]. TT refers to the sum of the combined and uncombined T, and FT refers to the uncombined part of the body circulation, both of which are important biomarkers that need to be monitored in the training supervision and training evaluation of elite athletes [11,12,13,14,15,16] because they are widely used early indicators of an imbalance between anabolic and catabolic metabolism [12]. FT is referred to as the physiologically active fraction because only the free form can go through the cell membrane to interact with the androgen receptor, which makes it physiological significant. Therefore, FT could improve precision in the assessment of androgenic status compared with TT. Nevertheless, TT and FT present a dynamic equilibrium state in the human body, so the level of TT can not only reflect the reserve of testosterone in the body, but also jointly reflect the athlete’s physical state with the ratio of other biomarkers such as cortisol, estrogen, etc. [3]. Thus, specific analysis methods with adequate accuracy and sensitivity for TT and FT need to be developed.

Similar to clinical laboratories, TT was measured by immunoassays in sport scientific research laboratories extensively [17,18,19,20,21]. It is well known that immunoassays lack specificity and thus directly affect measurement accuracy. So far, FT measurement is still a thorny issue whether directly determined by an immunoassay or calculated by using TT, albumin, and SHBG content with algorithms based on incorrect mathematical models of T binding to SHBG [8,22].

LC-MS/MS, as an advanced analytical technique, has been widely used in various fields for quantitative and qualitative measurements due to its high sensitivity and specificity. Recently, more and more LC-MS/MS determination methods for serum TT or FT with different sample preparation techniques have been reported [23,24,25,26,27,28,29,30,31,32,33,34,35]. Almost all of these studies can only determine TT [23,24,25,26,27,28,29,30,31,32,33]. There are only a few available articles that can simultaneously measure TT and FT in serum by LC-MS/MS [34,35]. However, these methods involve dialysis and derivatization procedures to prepare FT, which require at least 6 to 12 h for the sample pretreatment. So, these methods are time-consuming and reagent-consuming.

In the field of athlete training monitoring, firstly, the test results need to be fed back to coaches and scientific researchers as soon as possible, so as to adjust the training plan accordingly. This requires the determination method to be both accurate and rapid. In addition, circulating FT levels in women are very low due to significant gender and individual differences in T concentration. Therefore, it is necessary to establish a fast, accurate method with a wide enough linear range to monitor TT and FT. In order to tackle the practical problem, we developed and validated a simple, sensitive, and accurate LC-MS/MS method with an adequately wide linearity range for simultaneously determining TT and FT in this paper. For TT, only one LLE step was required. Compared to TT pretreatment, a protein removal step of a 20 min ultrafiltration prior to LLE for FT was needed. This method could significantly improve work efficiency and reduce sample preprocessing time, satisfying the demands of training monitoring for athletes. Finally, this point was effectively verified by the practical application of using this method on a large-scale serum sample of 200 professional athletes for the first time.

## 2. Results and Discussion

### 2.1. Separation of FT from TT

The measurement of FT is still challenging because a simple and fast pretreatment method has not been found. A dialysis method was adopted in previously published articles to extract and purify FT from serum [34,35,36], which required a significant amount of time, usually several hours or even dozens of hours. The key issue influencing the determination of serum FT is the separation of the protein-binding fraction and unbinding fraction (FT). Thus, removing proteins from the serum matrix is the primary problem to be solved. Ultrafiltration, as an extraction method based on membrane materials, the molecular weight cutoff, and centrifugation conditions that can separate substances of different molecular weights, is well known for its time efficiency. Several published articles have used ultrafiltration to separate free fractions from protein-binding steroids [37,38], proving that ultrafiltration is a simple and reliable method for extracting free steroids. Here, we used an ultrafiltration method in a simple physical manner to achieve the separation of FT and the protein-binding fraction, including SHBG-, HAS-, CBG-, and ORM-bound forms, only by about 20 min. Considering SHBG with a molecular weight of approximately 90 kDa [8], HSA with a molecular weight of approximately 66k Da [39], CBG with a molecular weight of approximately 58 kDa [40], ORM with a molecular weight of approximately 37–54 kDa [41], and β2 microglobulin (BMG) with a molecular weight of about 11 kDa, which is the smallest molecular weight protein in serum, a 10 kDa ultrafiltration tube was selected to achieve a better protein removal effect. Millipore 10 kDa ultrafiltration tubes were adopted in this study due to their vertical membrane structure, which can reduce concentration polarization, accelerate centrifugation speed, and greatly shorten the time required for centrifugation. The whole centrifugation procedure only takes about 20 min with an interception ratio of more than 95%. The interception ratio refers to the proportion of proteins with a molecular weight exceeding 10 kDa that are intercepted by the filter membrane. By using 10 kDa ultrafiltration tubes, almost all the protein-binding T and other interfering proteins in serum could be discarded, and FT was separated into the bottom ultrafiltrate consequently. Compared with previous published articles, the ultrafiltration tube used in this study has a higher molecular weight interception rate and smaller nonspecific adsorption, which can effectively improve the recovery, especially for low-concentration free steroids.

### 2.2. Method Validation

#### 2.2.1. Ultrafiltration for FT

No difference was found in the measurement results between filtration once and filtration twice (Table 1), indicating that the ultrafiltration membrane used in this study has no adsorption effect on FT. By evaluating the FT determination results of ultrafiltration after three different-level binding proteins (SHBG, HAS, and CBG) were equilibrated with different concentration levels of T, FT increased as the concentrations of the three binding proteins decreased (Table S1), which is consistent with the protein-binding theory of drugs. That is to say, the ultrafiltration method established in this study can accurately determine the amount of FT.

#### 2.2.2. Specificity

Quantitative ions of T (288.80 → 109.20) and T-C13 (291.90 → 112.10) were investigated by comparing a blank serum matrix spiked with T and T-C13 at a low quality control (LQC, 0.05 ng/mL) level, real human serum sample with a concentration similar to the LQC level, and blank serum matrix to estimate the specificity. No potential interfering substances at retention times of T and T-C13 were found in the above three kinds of matrices (Figure S1) and method specificity was preferred.

#### 2.2.3. Linearity and Lower Limit of Quantitation (LLOQ)

Eight levels of calibrators from 0.02 ng/mL to 100 ng/mL were used to generate the calibration curves. The results showed excellent linearity with correlation coefficients (R2) of greater than 0.99 between 0.02 ng/mL and 100 ng/mL. A representative calibration curve and other details of calibration curves during method validation are presented in Figure S2 and Table S2 in Supplementary Materials, respectively. LLOQ refers to the lowest concentration point in the linear range, which was 0.02 ng/mL and RSD < 20%.

#### 2.2.4. Accuracy and Precision

As shown in Table 2, accuracy ranged from 90.0 to 112.7%; intra-batch and inter-batch precisions (RSD) were between 1.6 and 8.4%. Accuracy and intra-batch and inter-batch precisions for all three level concentrations including low quality control (0.05 ng/mL, LQC), medium quality control (10 ng/mL, MQC), and high quality control (80 ng/mL, HQC) levels were within the limits presented in the published guidelines [42,43]. More details are found in Table S3.

#### 2.2.5. Recovery

Recoveries at three concentration levels were evaluated with the results of LQC within 85.6–109.8%, MQC within 75.1–93.7%, and HQC within 74.1–81.3% in a five-replicate manner. Results of recovery are presented in Table S4.

#### 2.2.6. Matrix Effect

The matrix effect is one of the challenges encountered in the current application of LC-MS/MS technology. The presence of interfering components in biological sample extracts has a significant impact on the mass spectrometry signal of analytes. Ion suppression and ion enhancement are two forms of the matrix effect, which are manifested as the reduction and enhancement in an analyte signal by matrix components, with ion suppression being more common [44,45]. 

Serum contains dissolved proteins and other materials, which can interfere with charge transfer during the ionization process in the gas phase between LC and MS [46]. The matrix effect may not always be completely circumventable because a perfectly consistent matrix does not exist, but it can be significantly minimized and largely compensated for by various approaches, such as standard addition, matrix-matched calibration, dilution, and the use of isotopic analogs of the analytes as internal standards [47]. In this study, a 13C-labeled isotope internal standard and matrix-matched calibration were both utilized to minimize the matrix effect. Consequently, a tiny matrix effect was observed at different concentration levels from 96.0 to 97.8% as shown in Table 3.

#### 2.2.7. Stability of Extracts

Considering the large number of batch testing samples, the prepared samples might wait for a certain period of time in the sequence in the automatic sampler for injection. In practical work, automatic sampler temperature of different LC-MS/MS instruments could be controlled to 4 °C or room temperature. Therefore, the stabilities of extracts after being placed at 4 °C and room temperature for 24 h were investigated, respectively. Accuracy ranged from 92.3% to 93.8% after 24 h of storage at 4 °C (Table S5). Accuracy ranged from 95.5% to 103.0% after 24 h of storage at room temperature (Table S6).

### 2.3. Application for Real Serum Samples of Elite Athletes

In the field of sport science, OTS can cause a decrease in athletic performance, which is an emerging disorder resulting from an excessive training load coupled with inadequate recovery and poor-quality sleep [5]. For competitive athletes, successful training not only involves overload but also avoids the combination of excessive overload with inadequate recovery [48]. Therefore, it is especially important to recognize the OTS as early as possible. One efficient and reliable approach is to monitor and measure biomarkers of athletes. Positive changes in basal T levels are associated with increases in lean mass and strength [49]; it is reasonable to conclude that the alteration of the T level is positively correlated with human sport performance. Moreover, from the previously published literature, decreasing serum TT and FT have been considered as indicators of the overtrained state [4,5,48,49,50,51]. Thus, accurately and quickly measuring TT and FT are extremely necessary for sports training.

**Figure 1:**
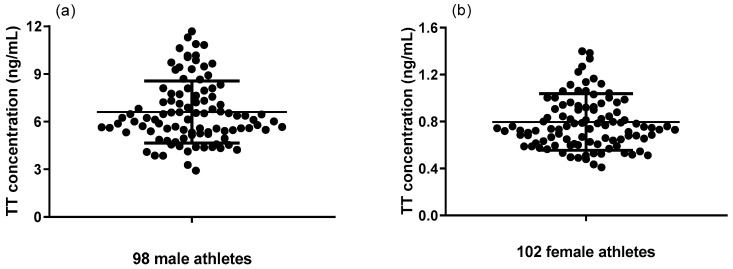
Serum TT levels of (**a**) 98 male athletes and (**b**) 102 female athletes.

**Figure 2:**
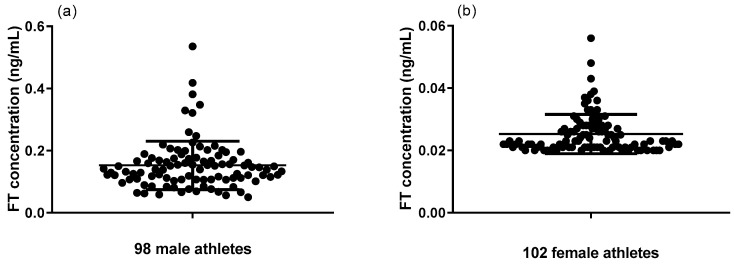
Serum FT levels of (**a**) 98 male athletes and (**b**) 102 female athletes.

**Figure 3:**
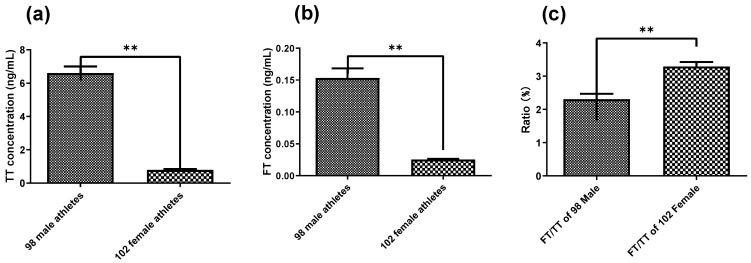
Difference of (**a**) TT between male and female professional athletes and (**b**) FT between male and female professional athletes, and (**c**) FT to TT ratios of 98 male athletes and 102 female athletes (independent two-tailed *t* test, ** *p* < 0.001).

**Figure 4:**
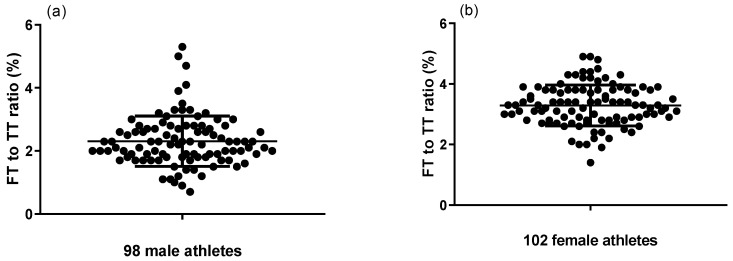
FT-to-TT ratios of (**a**) 98 male athletes and (**b**) 102 female athletes.

Utilizing this newly developed simple LC-MS/MS method, both serum TT and FT were evaluated for 200 Chinese national shooting athletes (98 males and 102 females). Serum TT levels of 98 male athletes and 102 female athletes ranged from 2.91 to 11.68 ng/mL and 0.41 to 1.62 ng/mL, respectively (Figure 1). Serum FT levels of 98 male athletes and 102 female athletes ranged from 0.05 to 0.54 ng/mL and 0.02 to 0.06 ng/mL, respectively (Figure 2). The differences between male and female athletes were compared, which are shown in Figure 3a,b. As expected, TT and FT levels of male athletes were significantly higher than the female group. In addition, elite athletes had significantly higher TT and FT upper limit levels compared to the general population (*p* < 0.001). This is mainly because endurably physical exercise could improve FT and TT levels, irrespective of moderate or intense physical activity [49,50,51]. In fact, endurance exercise increases both FT and TT levels by upregulating FT levels in an SHBG binding affinity-independent manner [51]. As can be seen from Equation (1), the increase in the FT level will directly lead to the increase in the FT/TT ratio. Consequently, FT/TT ratios of professional athletes were conspicuously higher than the upper limit of the general population range, regardless of gender (Figure 4). The FT/TT ratio of female athletes is higher than that of male athletes (Figure 3c), which may be due to women’s lower basal testosterone levels, and the increase in testosterone levels caused by exercise is more significant.

(1) $$\frac{FT}{TT}\left(\%\right)=\frac{FT}{FT+CombindedT}\times 100\%$$

**Figure 5:**
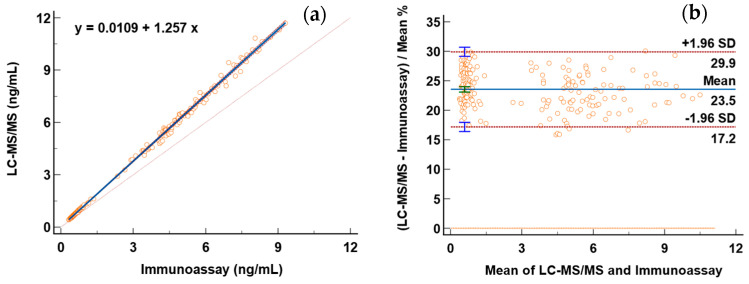
Comparison between LC-MS/MS and immunoassay methods. (**a**) Passing and Bablok regression analysis. (**b**) Bland-Altman plot.

In order to compare the newly established LC-MS/MS method and the original chemiluminescence immunoassay method in our laboratory, both of the two methods were used to measure TT (only serum TT can be measured by a chemiluminescence immunoassay in our laboratory initially) in serum samples from 200 professional athletes. The linear regression plots for the comparison study using Passing and Bablok non-parametric linear regression are displayed in Figure 5a. The resulting regression line was y = 0.0109 (95% CI: −0.0019 to 0.0211) + 1.257 (95% CI: 1.2451 to 1.2699) x. However, the Pearson correlation coefficient (R^2^) showed that these methods were highly correlated, R^2^ = 0.9976, *p* < 0.0001. The methods were also compared using the Bland–Altman plot (Figure 5b). In the percentage plot, the relative bias was 23.5%, and the limits of agreement ranged from 17.2 to 29.9% for the proportional difference. It indicated that the measurement results from these two methods were highly correlated but with systematic bias. The results obtained by the LC-MS/MS method are 17.2–35.4% higher than those of the immunoassay, which is consistent with previously published research [30].

## 3. Materials and Methods

### 3.1. Materials and Ethical Approval

T with a purity of 99.3% and T-2,3,4-13C3 (13C 99.4%) with a purity of 99.5% were both purchased from Alta Scientific Co., Ltd. (Tianjin, China). Hexane (HPLC grade), ethyl acetate (HPLC grade), methanol (HPLC grade), and formic acid (HPLC grade) were supplied by Dikma Technologies Inc. (Beijing, China). Ultrapure water was obtained from a Milli-Q ultrapure water system (Millipore, MA, USA). Blank serum for method validation was purchased from Innoreagents (Huzhou, China). The ultrafiltration tube (0.5 mL/10 KD) was supplied by Merck (Millipore, MA, USA).

Athlete serum samples from shooting athletes of the Chinese national team were stored at −80 °C until use. The study was approved by the Research Ethics Committee of the China Institute of Sport Science (20220928).

### 3.2. Instrumentation and Analytical Conditions

The determination of T and T-13C3 was performed on a SHIMADZU 8060 LC-MS/MS system (Shimadzu, Kyoto, Japan) using a column of Agilent Poroshell 120 EC-C18, 2.1 × 100 mm, 2.7 µm (Agilent, Santa Clara, CA, USA), maintained at 40 °C. Mobile phases consisted of 0.1% formic acid in water (A) and 0.1% formic acid in methanol (B). The injected samples were eluted using a gradient elution at a flow rate of 0.3 mL/min as follows: 0.0–0.1 min, 40% B; 0.1–5.0 min, linear from 40 to 70% B; 5.0–5.1 min, 98% B; 5.1–8.0 min, maintain at 98% B; and 8.0–10.0 min, linear from 98 to 40% B (Table S7). The autosampler was used with an injection volume of 10 μL. Quantitative ion transitions of T were then scanned by multiple reaction monitoring in the positive electrospray ionization mode. Nitrogen was used as nebulizer gas, drying gas, and collision gas. The MS conditions for the analysis were as follows: nebulizer gas, 2.8 L/min; drying gas, 10 L/min; and interface temperature, 300 °C; DL temperature and heat block temperature were maintained at 250 °C and 400 °C, respectively. Quantitative ion transitions were m/z 288.80 → 109.20 (collision energy: 24 eV) for T and m/z 291.90 → 112.10 (collision energy: 27 eV) for T-13C3, respectively.

### 3.3. Preparation of Standard Solutions

A stock solution of T at 1 mg/mL was prepared in methanol. Working solutions of T with concentrations of 10,000 ng/mL, 8000 ng/mL, 4000 ng/mL, 1000 ng/mL, 100 ng/mL, 10 ng/mL, 5 ng/mL, and 2 ng/mL were prepared by diluting the stock solution in methanol. The purchased commercialized store solution of T-13C3 (internal standard, IS) was in acetonitrile with a concentration of 10 μg/mL. The IS working solution was diluted in a 1:1 (*v*:*v*) mixed solution of hexane and ethyl acetate at 0.5 ng/mL. All stock and working solutions were stored at −20 °C. Blank serum samples (99 µL) were spiked with a 1 µL T working solution to generate calibration curves with 8 different concentrations at 100 ng/mL, 80 ng/mL, 40 ng/mL, 10 ng/mL, 1 ng/mL, 0.1 ng/mL, 0.05 ng/mL, and 0.02 ng/mL. QC samples including high QC, medium QC, and low QC for T were 80 ng/mL, 10 ng/mL, and 0.05 ng/mL.

### 3.4. Sample Preparation of TT

One hundred microliters of the serum sample was transferred into a 1.5 mL polypropylene tube. Five hundred microliters of a 1:1 (*v*:*v*) mixture of hexane/ethyl acetate containing 0.5 ng/mL of T-13C3 was added to the sample tube, vortex-extracted for 30 s, and centrifuged at 10,000 r.p.m for 5 min. The supernatant was transferred to another clean tube and evaporated to dryness by N2. The dry residue was redissolved in a 100 μL initial proportion of the mobile phase, and 10 μL of the solution was injected into the LC–MS/MS system.

### 3.5. Sample Preparation of FT

Two hundred microliters of the serum sample was transferred into a 0.5 mL/10 KD ultrafiltration tube and centrifuged at 13,000 r.p.m for 20 min. After ultrafiltration, 100 μL separated serum was pretreated by one-step LLE as aforementioned in the “Sample Preparation of TT” section.

### 3.6. Method Validation

In accordance with widely recognized bioanalytical method validation guidelines [39,40], this method was fully validated by specificity, linearity, LLOQ, accuracy, precision, recovery, the matrix effect, and stability. Additionally, the quantitative collection of FT by the ultrafiltration membrane was also evaluated.

#### 3.6.1. Ultrafiltration for FT

The nonspecific adsorption of FT by the ultrafiltration membrane was investigated by comparing the measurement results of FT in serum samples from ten different athletes (five female and five male) with a once-filtered filtrate and twice-filtered filtrate during which a new membrane was used in the second round of ultrafiltration. In order to assess the accuracy of the ultrafiltration tube for the collection of FT, three different levels of SHBG (20 nmol/L, 80 nmol/L, 150 nmol/L), HAS (20 g/L, 40 g/L, 80 g/L), and CBG (10 mg/L, 20 mg/L, 30 mg/L) were spiked into blank serum matrices without proteins, and then T was spiked into each of them to make three different levels at 2 ng/mL, 10 ng/mL, and 50 ng/mL, respectively. Before ultrafiltration, all the samples were incubated at 37 °C for 1 h. 

#### 3.6.2. Specificity

Method specificity was to confirm if there were any interferences from the matrix or other substances present in the sample, which was carried out by analyzing blank matrix samples and blank samples spiked with reference standard solutions of T and IS. 

#### 3.6.3. Linearity and LLOQ

Method linearity was investigated by the analysis of calibration curve triplicates. Calibration curves were generated by plotting the peak area ratio of the analyte to IS against concentrations using weighted (1/×2) linear least-squares regression. LLOQ is defined as the concentration at which the quantitative ion signal-to-noise ratio is greater than 10 and should meet a relative standard deviation of less than 20% of at least 5 replicates. 

#### 3.6.4. Accuracy and Precision

Accuracy and precision were assessed by the determination of QC samples (n = 5 per level) at three levels (LQC, MQC, and HQC) on three consecutive days. Accuracy was expressed as the percentage of the measured value to true value, which should be within the range of 85–115% (LQC could be within the range of 80–120%). Precision was expressed as relative standard deviation (RSD), which should be no more than 15% (LQC could be no more than 20%). 

#### 3.6.5. Recovery

Recovery of T was evaluated by comparing the peak area ratio of the analyte to IS in QC samples at three levels (0.05 ng/mL, 10 ng/mL, and 80 ng/mL) with blank matrices spiked with the analyte prior to extraction with the peak area ratio of the analyte to IS spiked post-extraction of blank matrices. 

#### 3.6.6. Matrix Effect

In the bioanalytical LC–MS/MS method, the matrix effect is a very important validation parameter [37,38,39]. In this study, the effect was evaluated on ion suppression or enhancement with LQC, MQC, and HQC, which was calculated by the ratio of the peak area in the presence of the blank matrix to the peak area in the absence of the matrix by analyzing 5 replicates at each QC concentration level. 

#### 3.6.7. Stability of Extracts

The extracts of LQC, MQC, and HQC were retained and analyzed for 24 h at 4 °C and room temperature to estimate stability, respectively. 

### 3.7. Application to Real Athlete Serum Samples

Intravenous blood samples were collected by vacuum tubes with separation gel and coagulation from male and female athletes in fasting states randomly. All the serum samples were measured in replicates. TT and FT of 200 serum samples of shooting athletes from the Chinese national team were analyzed and monitored using this LC-MS/MS method, respectively. Data were processed by Shimadzu LabSolutions software (Version 5.89).

### 3.8. Statistical Analysis

The statistical analysis of the data from the comparison of LC-MS/MS and chemiluminescence immunoassay methods was performed by using MedCalc Software (Version 23.0.5).

An unpaired 2-tailed t test was performed to analyze the other data by using GraphPad Prism Software (Version 8.0.1).

## 4. Conclusions

A simple and rapid LC-MS/MS method for simultaneous measurement of TT and FT in human serum was established and fully validated. Compared with the previous reported preparation procedures involving dialysis, derivatization, solid phase extraction, or multi-step LLE, the newly developed method only required one-step LLE for TT and a 20 min ultrafiltration followed by one-step LLE for FT. It is very efficient and time-saving and could effectively meet the needs of physiological monitoring, screening OTS as early as possible in sports training. 

Finally, this method was used to study 200 elite national shooters, including 98 male athletes and 102 female athletes. To our knowledge, this is the first large scale evaluation of serum TT and FT in professional athletes. Serum TT and FT from different genders were investigated, respectively. Serum TT, FT, and FT/TT levels of professional athletes were significantly higher than those of the general population, as long-term endurance exercise could increase FT testosterone levels with a mediation by the sympathetic stimulation of T-secreting organs [51]. In addition, the newly developed LC-MS/MS method was compared with the original chemiluminescence immunoassay method in our laboratory by a measurement of serum TT. Similar to the previous report, serum TT levels determined by the LC-MS/MS method were 17.2–35.4% higher than the chemiluminescence immunoassay method. In order to diagnose OTS for a wider range of elite athletes by providing TT and FT levels during sports training as early as possible, it is necessary to investigate TT and FT levels for more athletes in different sports events by using this method.

## Figures and Tables

**Table 1:** Nonspecific adsorption of FT by ultrafiltration membrane.

	Female					Male				
	No. 1	No. 2	No. 3	No. 4	No. 5	No. 1	No. 2	No. 3	No. 4	No. 5
Filtered once (ng/mL)	0.038	0.047	0.098	0.143	0.211	0.121	0.206	0.452	0.559	0.884
Filtered twice (ng/mL)	0.021	0.059	0.071	0.167	0.182	0.139	0.172	0.401	0.522	0.829
RSD (%)	1.2	0.8	1.9	1.7	2.1	1.3	2.4	3.6	2.6	3.9

**Table 2:** Accuracy and precision of testosterone.

Spiked (ng/mL)	Accuracy (%) (n = 15)	Precision (%) Intra-Batch			Inter-Batch (n = 15)
		Batch 1 (n = 5)	Batch 2 (n = 5)	Batch 3 (n = 5)	
0.05	90.0–111.0	6.7	8.4	7.5	7.0
10	94.5–112.7	6.8	1.6	3.4	5.9
80	93.0–105.9	4.8	4.2	5.0	4.9

**Table 3:** Matrix effect of testosterone in serum.

LQC (0.05 ng/mL) (%)	Avg. (%)	RSD (%)	MQC (10 ng/mL) (%)	Avg. (%)	RSD (%)	HQC (80 ng/mL) (%)	Avg. (%)	RSD (%)
94.0	96.7	6.9	96.0	97.8	4.3	95.5	96.0	5.9
102.0			92.6			98.2		
102.2			98.3			103.7		
86.4			104.2			88.1		
99.0			97.7			94.4		

## Data Availability

The data presented in this study are available on request from the corresponding author due to privacy.

## Supplementary Materials

The following supporting information can be downloaded at: https://www.mdpi.com/article/10.3390/molecules29215007/s1, Figure S1: Specificity of method (A: Blank matrix spiked with testosterone and IS; B: Real human serum spiked with IS; C: Blank matrix). Figure S2: Representative calibration curve of testosterone. Table S1: Nonspecific adsorption of FT to the ultrafiltration membrane. Table S2: Quantitative results of FT with three different levels of SHBG, HAS and CBG. Table S3: Calibration curves and linearity information. Table S4: Accuracy and precision of testosterone. Table S5: Recoveries of testosterone at LQC, MQC and HQC (n = 5). Table S6: Stability of extracts at 4 °C after 24 h of storage. Table S7: Stability of extracts at room temperature after 24 h of storage. Table S8: Gradient elution of testosterone and IS.
