# Supplementary material for: Rapid Liquid Chromatography–Tandem Mass Spectrometry Method for Determination of Total and Free Testosterone in Human Serum and Its Application to Monitoring Biomarker Response of Elite Athletes
Source: Molecules. 2024 Oct 23;29(21):5007. doi: 10.3390/molecules29215007 (PMC11547523; doi:10.3390/molecules29215007)
Supplement: Supplementary file 1 [file molecules-29-05007-s001.zip › molecules-3259545-supplementary.pdf]

Supplementary Materials of
"A rapid LC-MS/MS method for determination of total and free testosterone in human serum and its application to monitoring biomarker response of elite athletes"

Jianli Zhang: China Institute of Sport Science, General Administration of Sport of China
Hang Yu: State Key Laboratory of Bioactive Substance and Function of Natural Medicines, Institute of Materia Medica, Chinese Academy of Medical Sciences & Peking Union Medical College
Yulin Shen: China Institute of Sport Science, General Administration of Sport of China
Xingya Yang: China Institute of Sport Science, General Administration of Sport of China
Yan Wang: State Key Laboratory of Bioactive Substance and Function of Natural Medicines, Institute of Materia Medica, Chinese Academy of Medical Sciences & Peking Union Medical College

Corresponding author: Jianli Zhang
Email address: zhang_jianli@sohu.com

Table S1. Nonspecific adsorption of FT to the ultrafiltration membrane.

	Female					Male				
	No.1	No.2	No.3	No.4	No.5	No.1	No.2	No.3	No.4	No.5
Once filtered (ng/mL)	0.038	0.047	0.098	0.143	0.211	0.121	0.206	0.452	0.559	0.884
Twice filtered (ng/mL)	0.021	0.059	0.071	0.167	0.182	0.139	0.172	0.401	0.522	0.829
RSD (%)	1.2	0.8	1.9	1.7	2.1	1.3	2.4	3.6	2.6	3.9

Table S2. Quantitative results of FT with three different levels of SHBG, HAS and CBG.

Spiked		2 ng/mL	10 ng/mL	50 ng/mL
SHBG	20 nmol/L	0.32 ng/mL	5.16 ng/mL	26.72 ng/mL
	80 nmol/L	0.09 ng/mL	1.22 ng/mL	8.53 ng/mL
	150 nmol/L	0.06 ng/mL	0.84 ng/mL	5.05 ng/mL
HAS	20 g/L	0.42 ng/mL	7.52 ng/mL	34.86 ng/mL
	40 g/L	0.18 ng/mL	3.03 ng/mL	18.61 ng/mL
	60 g/L	0.11 ng/mL	1.85 ng/mL	10.52 ng/mL
CBG	10 mg/L	1.65 ng/mL	8.01 ng/mL	41.85 ng/mL
	20 mg/L	1.23 ng/mL	6.65 ng/mL	30.25 ng/mL
	30 mg/L	0.98 ng/mL	4.52 ng/mL	22.52 ng/mL

Table S3. Calibration curves and linearity information.

concentration (ng/mL)	Testosterone					
	1	2	3	mean (ng/mL)	SD (ng/mL)	CV (%)
0.02	0.019	0.021	0.020	0.020	0.001	5.000
0.05	0.053	0.049	0.049	0.050	0.002	4.588
0.1	0.104	0.092	0.114	0.103	0.011	10.660
1	1.057	1.107	1.137	1.100	0.040	3.673
10	9.558	10.526	9.606	9.897	0.546	5.512
40	43.520	44.730	37.290	41.847	3.992	9.540
80	71.712	76.313	71.034	73.020	2.872	3.933
100	93.145	92.230	98.769	94.715	3.541	3.738
r	0.996942	0.993944	0.995101	0.994523	-	-

Table S4. Accuracy and precision of testosterone.

	Added (ng/mL)		Accuracy(%)	Mean(ng/mL)	RE(%)	RSD(%)
Batch-1	0.05	0.0495	99.000	0.050	-0.200	6.737
		0.0515	103.000			
		0.0545	109.000			
		0.0485	97.000			
		0.0455	91.000			
	10	9.686	96.860	9.985	-0.146	6.772
		9.668	96.680			
		9.452	94.520			
		9.972	99.720			
		11.149	111.490			
	80	76.042	95.053	77.730	-2.838	4.822
		76.605	95.756			
		74.438	93.048			
		84.144	105.180			

		77.419	96.774			
		0.0555	111.000			
		0.0530	106.000			
	0.05	0.0450	90.000	0.050	0.400	8.351
		0.0475	95.000			
		0.0500	100.000			
		11.084	110.840			
		10.780	107.800			
Batch-2	10	11.055	110.550	11.068	10.678	1.636
		11.271	112.710			
		11.149	111.490			
		83.018	103.773			
		84.296	105.370			
	80	84.707	105.884	82.000	2.500	4.206
		81.803	102.254			
		76.177	95.221			
		0.0535	107.000			
		0.0550	110.000			
	0.05	0.0470	94.000	0.051	1.000	7.508
		0.0505	101.000			
		0.0465	93.000			
		9.913	99.130			
		10.781	107.810			
Batch-3	10	10.587	105.870	10.512	5.118	3.475
		10.459	104.590			
		10.819	108.190			
		84.012	105.015			
		83.220	104.025			
	80	76.740	95.925	79.814	-0.232	4.985
		74.868	93.585			
		80.231	100.289			
	0.05			0.050	0.400	7.021
inter-day	10			10.522	5.217	5.916
	80			79.848	-0.190	4.881

Table S5. Recoveries of testosterone at LQC, MQC and HQC (n=5).

LQC (0.05ng/mL) (%)	Average Recovery (%)	RSD (%)	MQC (10ng/mL) (%)	Average Recovery (%)	RSD (%)	HQC (80ng/mL) (%)	Average Recovery (%)	RSD (%)
105.4			81.8			74.1		
87.0			86.6			77.6		
85.6	95.6	10.4	82.4	83.9	8.1	73.6	77.2	4.3
90.2			75.1			81.3		
109.8			93.7			79.4		

Table S6. Stability of extracts at 4°C after 24h of storage.

Added (ng/mL)	before	after	Accuracy(%)	Mean(ng/mL)	RSD(%)
0.05	0.0500	0.0530	106.000	93.848	9.429
	0.0505	0.0440	87.129		
	0.0450	0.0430	95.556		
	0.0515	0.0430	83.495		
	0.0510	0.0495	97.059		
10	10.231	9.657	94.390	94.822	4.941
	9.589	9.437	98.415		
	10.692	9.376	87.692		
	10.664	10.022	93.980		
	11.206	11.165	99.634		
80	84.044	75.213	89.492	92.337	4.269
	82.044	76.819	93.631		
	85.546	77.148	90.183		
	83.900	82.817	98.709		
	85.056	76.267	89.667		

Table S7. Stability of extracts at room temperature after 24h of storage.

Added (ng/mL)	before	after	Accuracy(%)	Mean	RSD(%)
0.05	0.050	0.047	94.000	97.351	6.546
	0.049	0.053	108.163		
	0.050	0.047	94.000		
	0.054	0.050	92.593		
	0.050	0.049	98.000		
10	9.149	9.359	102.295	103.029	3.598
	9.308	9.262	99.506		
	9.661	9.642	99.803		
	9.488	10.003	105.428		
	9.957	10.765	108.115		
80	79.976	72.363	90.481	95.548	3.960
	79.080	73.168	92.524		
	75.350	74.111	98.356		
	81.228	80.200	98.734		
	77.326	75.505	97.645		

Table S8. Gradient elution of testosterone and IS.

Time(min)	A(%)	B(%)
0.1	60	40
5.0	30	70
5.1	2	98
7.0	2	98
10.0	60	40

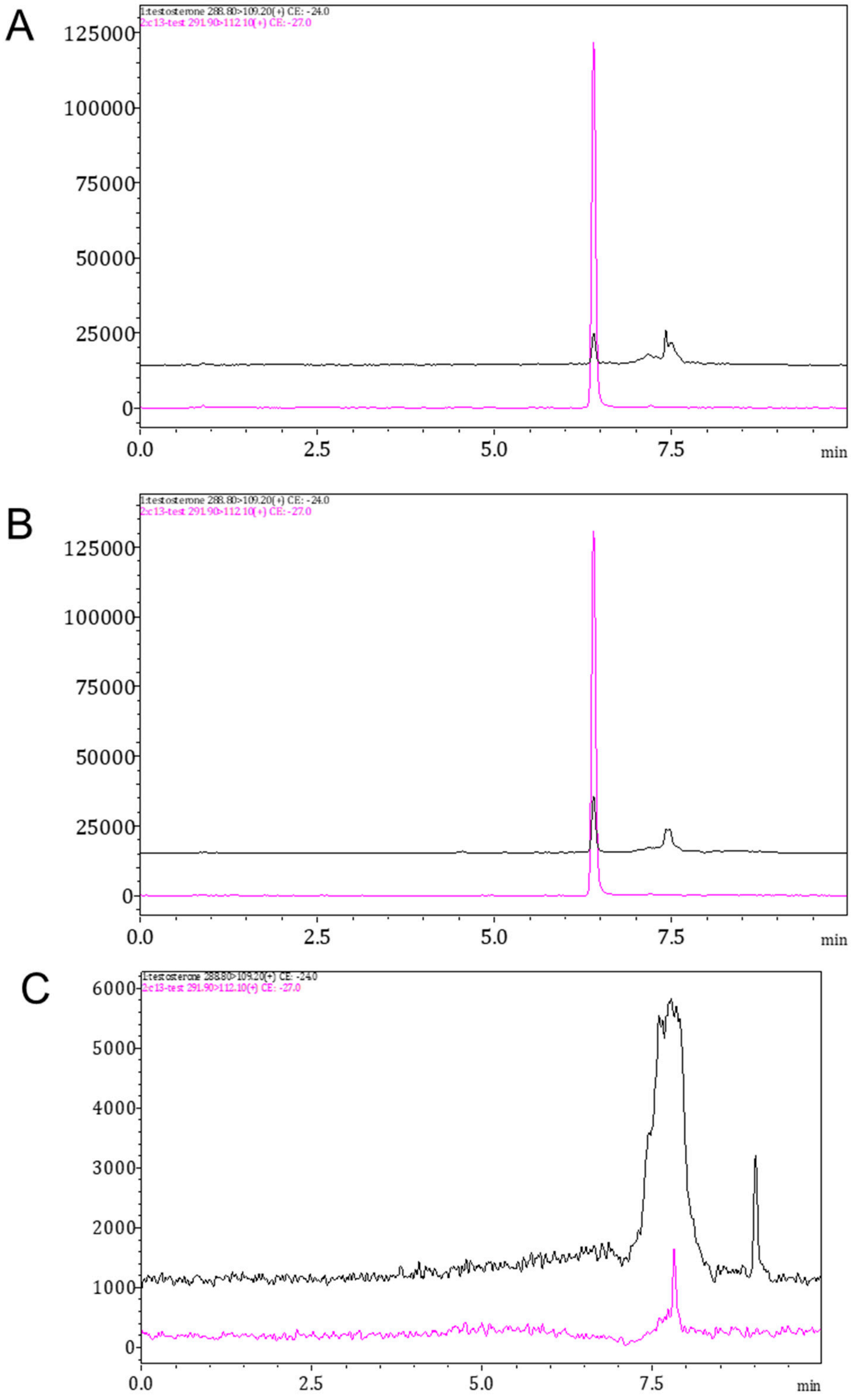

Figure S1. specificity of method (A: Blank matrix spiked with testosterone and IS; B: Real human serum spiked with IS; C: Blank matrix).

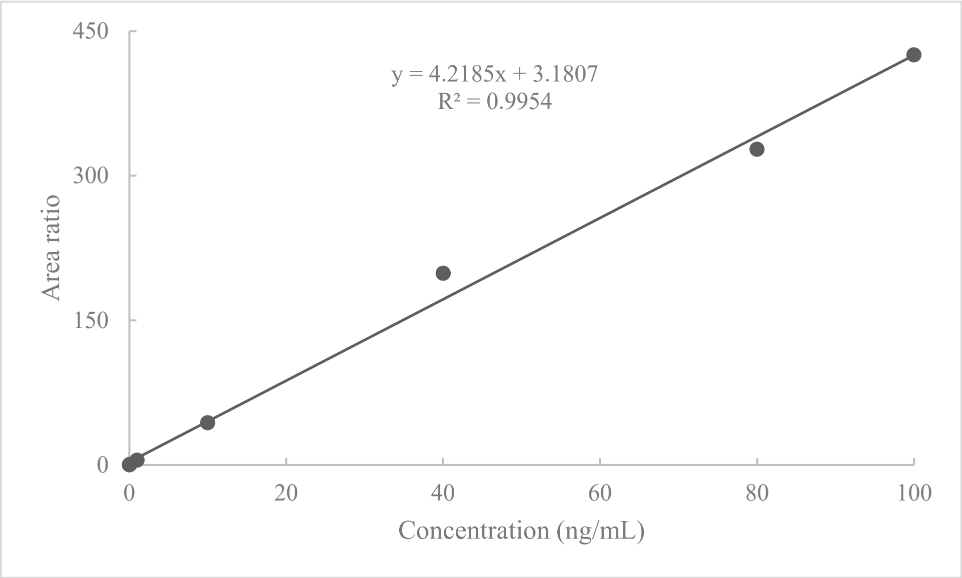

Figure S2. Representative calibration curve of testosterone.
